# Supplementary material for: Factors Determining the Agreement between Aerobic Threshold and Point of Maximal Fat Oxidation: Follow-Up on a Systematic Review and Meta-Analysis on Association
Source: Int J Environ Res Public Health. 2022 Dec 27;20(1):453. doi: 10.3390/ijerph20010453 (PMC9819531; doi:10.3390/ijerph20010453)
Supplement: Supplementary file 1 [file ijerph-20-00453-s001.zip › ijerph-2007193-supplementary/ijerph-2007193-supplementary.pdf]

Section S1: FAT_{max} ZONE

FAT_{max} zone was estimated using a meta-analytical approach by computing the distance, in terms of VO_2, between the Upper (X_2) and Lower (X_1) FAT_{max} zone range (Supplementary Material Section S1, Figure S1), which correspond to the acceptable range (to be divide equally in both sides of the FAT_{max}) in which the differences between FAT_{max} and AeT can fall and be considered within the FAT_{max} zone.

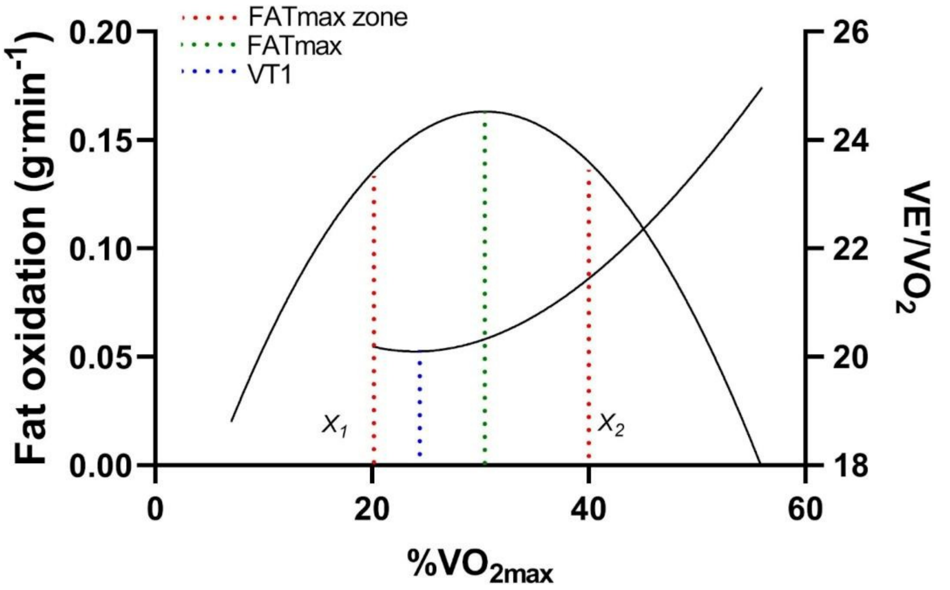

Figure S1. Fat oxidation and ventilatory equivalents of oxygen during a graded exercise test performed on a treadmill. The FAT_{max} (green dotted line) and the FAT_{max} zone (between red dotted lines) were determined by fitting a cubic

polynomial regression using the fat oxidation rate and the relative oxygen uptake at each stage of a test performed by a representative individual. In addition, the aerobic threshold (blue) was defined as the exercise intensity at which the ventilatory equivalents of oxygen breaks with linearity and showed a sustained increment. Lower (X_1) and upper (X_2) FAT_{max} zone range.

Six articles (Supplementary Material Section S1, Figure S2) reporting the FAT_{max} zone in trained (n=52) and untrained (n=70) individuals were identified from a total of 20 studies that performed a mathematical modelling of the fat oxidation kinetics [1]. The FAT_{max} zone range was calculated using Metafor package of R software (version 4.0.4) (The R Foundation, Vienna, Austria) by computing the mean difference (MD) between the Upper (X_1) (mean ± SD) and Lower (X_2) FAT_{max} zone intensity range (mean ± SD) of each study. Additionally, since Upper and Lower FAT_{max} zone intensities were paired data deriving from the same individual, the correlation between the two exercise intensities was accounted for MD calculation. Specifically, the correlation coefficient between Upper and Lower FAT_{max} zone intensities was estimated to be 0.96 for the included studies in the meta-analysis. When MDs were expressed in $\%VO_{2max}$, they were clustered in two studies; hence, a multilevel meta-analysis was performed to account for both the within-studies and between-studies variance [40,47]. Summary MD and *r* estimates were determined using a random-effects model and presented as mean and 95% confidence (CI) and prediction (PI) interval [40] (see Supplementary Material Section S1, Figure S2).

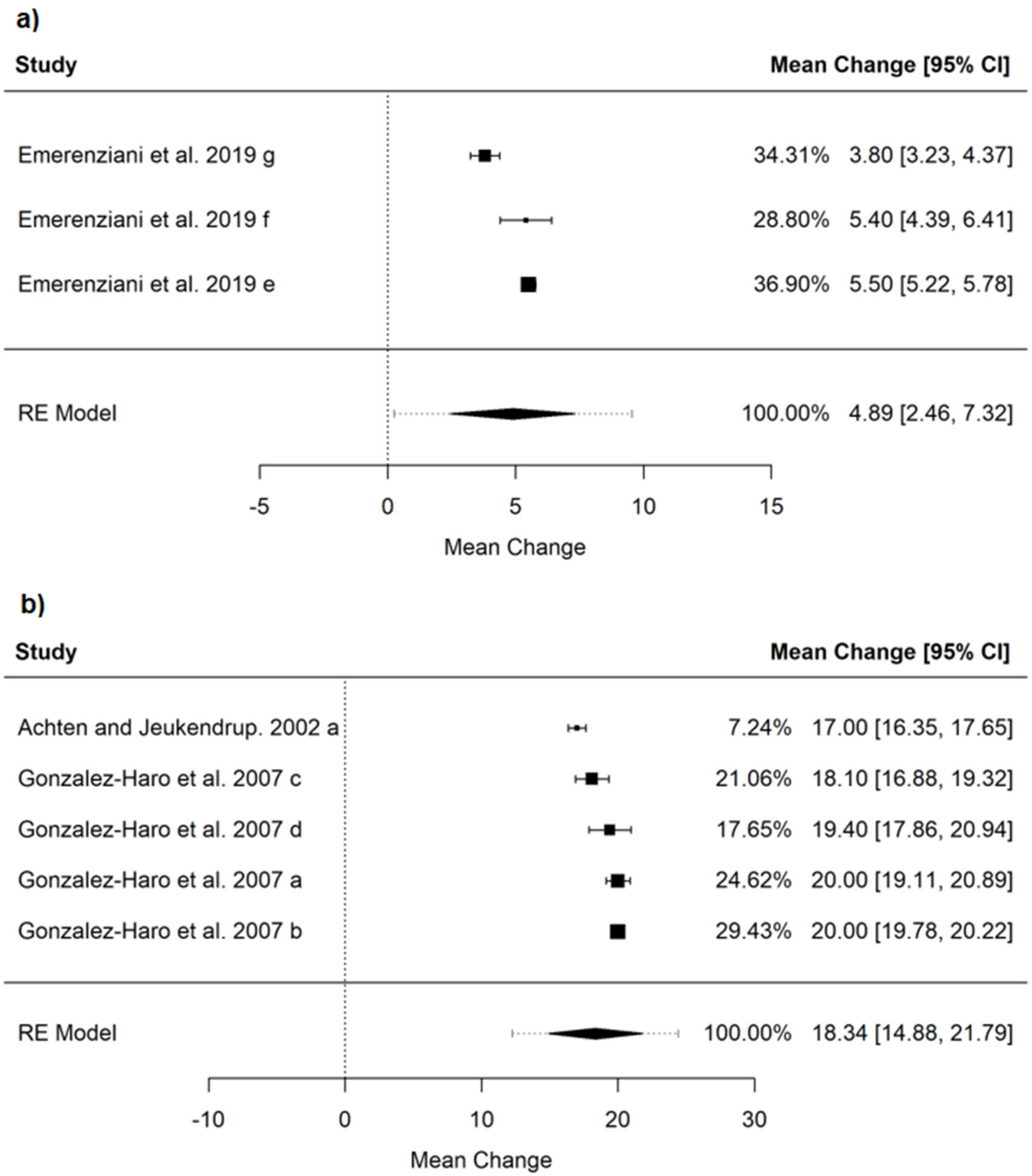
a)
Study
Mean Change [95% CI]
Emerenziani et al. 2019 g
34.31% 3.80 [3.23, 4.37]
Emerenziani et al. 2019 f
28.80% 5.40 [4.39, 6.41]
Emerenziani et al. 2019 e
36.90% 5.50 [5.22, 5.78]
RE Model
100.00% 4.89 [2.46, 7.32]
-5
0
5
10
15
Mean Change
b)
Study
Mean Change [95% CI]
Achten and Jeukendrup. 2002 a
7.24% 17.00 [16.35, 17.65]
Gonzalez-Haro et al. 2007 c
21.06% 18.10 [16.88, 19.32]
Gonzalez-Haro et al. 2007 d
17.65% 19.40 [17.86, 20.94]
Gonzalez-Haro et al. 2007 a
24.62% 20.00 [19.11, 20.89]
Gonzalez-Haro et al. 2007 b
29.43% 20.00 [19.78, 20.22]
RE Model
100.00% 18.34 [14.88, 21.79]
-10
0
10
20
30
Mean Change

Figure S2. Forest plots of the mean change (e.g., difference between Upper and Lower FAT_{max} zone limits) in the VO_2 (expressed as ml/kg/min (a panel) and $\%VO_{2max}$ (b panel)). The mean change corresponds to the exercise intensity range with fat oxidation higher than 90% of FAT_{max} which has to be divided equally in both sides of the FAT_{max}.

NOTE: Achten and Jeukendrup, 2002a (moderately trained cyclist); Emerenzianni et al. 2019e (women with obesity class I), Emerenzianni et al. 2019f (women with obesity class II), and Emerenzianni et al. 2019g (women with obesity class III); Gonzalez-Haro et al. 2007a (male triathletes), Gonzalez-Haro et al. 2007b (female triathletes), Gonzalez-Haro et al. 2007c (road cyclist) and Gonzalez-Haro et al. 2007d (male mountain bikers).

Section S2: AGREEMENT INDICATORS

Table S1.1. Estimation of 95% Limits of Agreement by subgroup for ml/min/kg method.

Subgroup	Subset	Studies	Sample	N	Bias	sd_bias	LOA_L	LOA_U	CI_L	CI_U
Gender	Male	7	9	326	-2.07	4.02	-9.96	5.82	-15.04	10.90
Gender	Female	3	4	186	-0.55	3.50	-7.42	6.31	-21.82	20.72
Physical level	Active	5	7	257	-2.28	6.15	-14.33	9.77	-22.68	18.12
Physical level	Inactive	5	6	255	-0.72	1.98	-4.60	3.15	-8.76	7.31
Ergometer	Cycle	6	8	348	-2.48	6.00	-14.24	9.28	-20.16	15.20
Ergometer	Treadmill	4	5	164	-0.21	1.53	-3.21	2.79	-6.29	5.87
AeT method	Lactate	5	7	322	-1.88	5.61	-12.87	9.11	-20.55	16.79
AeT method	Gas analysis	6	6	190	-1.33	3.08	-7.37	4.71	-11.76	9.10
FAT max method	Visual	7	10	416	-1.00	3.80	-8.44	6.45	-14.14	12.14
FATmax max method	Mathematical	3	3	96	-2.58	4.27	-10.94	5.78	-24.75	19.58
VO2max protocol	Short	10	11	434	-1.85	3.75	-9.19	5.49	-12.83	9.13
VO2max protocol	Long	2	2	78	5.06	9.82	-14.19	24.31	-89.00	99.13
FATmax protocol	Identical	9	12	490	-0.85	3.27	-7.25	5.56	-11.23	9.54

Subgroup	Subset	Studies	Sample	N	Bias	sd_bias	LOA_L	LOA_U	CI_L	CI_U
FATmax protocol	Additional	1	1	22	-6.70	4.08	-14.70	1.30		
All Studies	ml/min/kg	10	13	512	-1.49	3.89	-9.12	6.14	-12.89	9.90
All Studies	ml/min/kg (w/o outliers)	10	11	434	-1.85	3.75	-9.19	5.49	-12.83	9.13

Table S1.2. Estimation of 95% Limits of Agreement by subgroup for $\%VO_{2max}$ method.

Subgroup	Subset	Studies	Sample	N	Bias	sd_bias	LOA_L	LOA_U	CI_L	CI_U
Gender	Male	9	10	368	-5.87	9.91	-25.28	13.55	-35.09	23.35
Gender	Female	2	2	195	-8.70	13.90	-35.95	18.55	-164.15	146.74
Physical level	Active	6	6	156	-5.31	10.30	-25.50	14.88	-37.30	26.68
Physical level	Inactive	5	6	407	-7.58	10.49	-28.14	12.99	-46.03	30.88
Ergometer	Cycle	5	5	99	-6.83	10.63	-27.66	14.00	-41.92	28.27
Ergometer	Treadmill	6	7	464	-6.04	10.44	-26.50	14.42	-41.11	29.03
AeT method	Lactate	4	4	73	-6.95	10.60	-27.72	13.83	-46.67	32.78
AeT method	Gas analysis	8	8	490	-6.14	9.83	-25.41	13.12	-36.19	23.90
FAT max method	Visual	6	7	445	-5.29	10.73	-26.33	15.75	-41.52	30.94
FATmax max method	Mathematical	5	5	118	-7.62	10.12	-27.45	12.21	-39.07	23.82
VO2max protocol	Short	9	10	541	-5.15	9.96	-24.67	14.37	-34.25	23.95
VO2max protocol	Long	2	2	22	-12.10	10.47	-32.62	8.41	-165.00	140.80
FATmax protocol	Identical	10	11	541	-5.92	10.28	-26.06	14.23	-35.40	23.57
FATmax protocol	Additional	1	1	22	-11.30	8.78	-28.50	5.90		

Subgroup	Subset	Studies	Sample	N	Bias	sd_bias	LOA_L	LOA_U	CI_L	CI_U
All Studies	%VO2max	11	12	563	-6.39	10.24	-26.47	13.69	-35.03	22.25
All Studies	%VO2max (w/o outliers)	10	11	406	-5.12	9.21	-23.18	12.94	-31.30	21.06

Section S3: SENSITIVITY ANALYSIS

Table S2.1. Estimation of 95% Limits of Agreement by subgroup for ml/min/kg method.

Subgroup	Subset	Studies	Sample	N	Bias	sd_bias	LOA_L	LOA_U	CI_L	CI_U
Gender	Male	7	9	326	-1.57	3.67	-8.76	5.61	-13.63	10.48
Gender	Female	3	4	186	-0.58	3.49	-7.43	6.27	-21.91	20.74
Physical level	Active	5	7	257	-1.57	5.73	-12.80	9.66	-20.70	17.57
Physical level	Inactive	5	6	255	-0.85	2.01	-4.78	3.09	-8.90	7.21
Ergometer	Cycle	6	8	348	-1.90	5.72	-13.11	9.31	-18.75	14.95
Ergometer	Treadmill	4	5	164	-0.31	1.63	-3.51	2.88	-6.65	6.02
AeT method	Lactate	5	7	322	-1.41	5.18	-11.57	8.76	-19.26	16.45
AeT method	Gas analysis	6	6	190	-1.33	3.08	-7.37	4.71	-11.76	9.10
FAT max method	Visual	7	10	416	-0.64	3.41	-7.32	6.05	-13.28	12.01
FATmax max method	Mathematical	3	3	96	-2.58	4.27	-10.94	5.78	-24.75	19.58
VO2max protocol	Short	10	11	434	-1.89	3.74	-9.22	5.43	-12.85	9.07
VO2max protocol	Long	2	2	78	5.06	9.82	-14.19	24.31	-89.00	99.13
FATmax protocol	Identical	9	12	490	-0.61	3.05	-6.59	5.37	-10.68	9.46
FATmax protocol	Additional	1	1	22	-6.70	4.08	-14.70	1.30		
All Studies	ml/min/kg	10	13	512	-1.20	3.59	-8.24	5.84	-11.92	9.52
All Studies	ml/min/kg (w/o outliers)	10	11	434	-1.89	3.74	-9.22	5.43	-12.85	9.07

Table S2.2. Estimation of 95% Limits of Agreement by subgroup for %VO_{2max} method.

Subgroup	Subset	Studies	Sample	N	Bias	sd_bias	LOA_L	LOA_U	CI_L	CI_U
Gender	Male	9	10	368	-6.02	9.86	-25.33	13.30	-35.08	23.04
Gender	Female	2	2	195	-8.70	13.90	-35.95	18.55	-164.15	146.74
Physical level	Active	6	6	156	-5.31	10.30	-25.50	14.88	-37.30	26.68
Physical level	Inactive	5	6	407	-7.84	10.34	-28.11	12.44	-45.77	30.09
Ergometer	Cycle	5	5	99	-6.83	10.63	-27.66	14.00	-41.92	28.27
Ergometer	Treadmill	6	7	464	-6.26	10.36	-26.56	14.05	-41.04	28.53
AeT method	Lactate	4	4	73	-6.95	10.60	-27.72	13.83	-46.67	32.78
AeT method	Gas analysis	8	8	490	-6.14	9.83	-25.41	13.12	-36.19	23.90
FAT max method	Visual	6	7	445	-5.51	10.67	-26.43	15.41	-41.52	30.51
FATmax max method	Mathematical	5	5	118	-7.62	10.12	-27.45	12.21	-39.07	23.82
VO2max protocol	Short	9	10	541	-5.30	9.92	-24.74	14.14	-34.27	23.68
VO2max protocol	Long	2	2	22	-12.10	10.47	-32.62	8.41	-165.00	140.80
FATmax protocol	Identical	10	11	541	-6.05	10.23	-26.11	14.01	-35.40	23.29
FATmax protocol	Additional	1	1	22	-11.30	8.78	-28.50	5.90		
All Studies	%VO2max	11	12	563	-6.51	10.20	-26.50	13.47	-35.01	21.98

Subgroup	Subset	Studies	Sample	N	Bias	sd_bias	LOA_L	LOA_U	CI_L	CI_U
All Studies	%VO2max (w/o outliers)	10	11	406	-5.25	9.18	-23.24	12.74	-31.32	20.81
